# Serological and Molecular Investigation of *Brucella* Species in Dogs in Pakistan

**DOI:** 10.3390/pathogens8040294

**Published:** 2019-12-13

**Authors:** Tariq Jamil, Falk Melzer, Iahtasham Khan, Mudassar Iqbal, Muhammad Saqib, Muhammad Hammad Hussain, Stefan Schwarz, Heinrich Neubauer

**Affiliations:** 1 Institute of Bacterial Infections and Zoonoses, Friedrich-Loeffler-Institut, 07743 Jena, Germany; falk.melzer@fli.de (F.M.); heinrich.neubauer@fli.de (H.N.); 2 Institute of Microbiology and Epizootics, Freie Universität Berlin, 14163 Berlin, Germany; stefan.schwarz@fu-berlin.de; 3 Section of Epidemiology and Public Health, University of Veterinary and Animal Sciences, Lahore, sub-campus Jhang, 12-Km Chiniot Road, Jhang 35200, Pakistan; iahtasham.khan@uvas.edu.pk; 4 University College of Veterinary and Animal Sciences, The Islamia University of Bahawalpur, Bahawalpur 63100, Pakistan; mudassar.iqbal@iub.edu.pk; 5 Department of Clinical Medicine and Surgery, Faculty of Veterinary Science, University of Agriculture, Faisalabad 38000, Pakistan; drsaqib_vet@hotmail.com; 6 1 Vance Street, Bardia 2565, Australia; m.hammad.hussain@gmail.com

**Keywords:** Dogs, *Brucella abortus*, *Brucella canis*, zoonosis, Pakistan

## Abstract

Brucellosis is an important bacterial zoonosis caused by *B. abortus* and *B. melitensis* in Pakistan. The status of canine brucellosis caused by *B. canis* remains obscure. In total, 181 serum samples were collected from stray and working dogs in two different prefectures viz. Faisalabad (*n* = 87) and Bahawalpur (*n* = 94). Presence of antibodies against *B. canis* and *B. abortus/B. melitensis* was determined using the slow agglutination test (SAT) and ELISA, respectively. Real-time PCR was performed to detect and differentiate *Brucella* DNA at the species level. In Faisalabad, the serological prevalence was found to be 9.2% (8/87) and 10.3% (9/87) by SAT and ELISA, respectively. Only one of the ELISA positive samples (1.15%) yielded amplification for *B. abortus* DNA. In Bahawalpur, 63.8% (60/94) samples were found positive by SAT; however, none of the samples was positive by ELISA or by real-time PCR. Location, age (≥1 year) and body condition (weak) were found to be associated with *B. canis* infection, whereas presence of wounds was found to be associated with *B. abortus* infection only. These findings point towards a risk of transmission from dog to livestock and humans and vice versa. The study expects to draw the attention of concerned authorities towards infection prevention and animal welfare. This study warrants further epidemiological investigation on brucellosis in pet dogs and their owners. To the best of our knowledge, this is first ever report on *B. canis* and *B. abortus* in dogs in Pakistan.

## 1. Introduction

Brucellosis is a serious bacterial zoonosis caused by *Brucella (B.)* species. It affects a wide range of wild and domestic animals worldwide. Of the 12 accepted nomo-species of *Brucella*, at least *B. abortus* (primary host: *Bovidae*), *B. melitensis* (small ruminants), *B. suis* (pigs) and to some extent *B. canis* (*Canidae*) are known human pathogens [1,2]. In domestic animals, abortion, retained placenta, orchitis and rarely arthritis are the cardinal symptoms that result in serious economic losses to the livestock industry [3]. Brucellosis poses a significant emerging threat to public health owing to the lack of vaccines, limited treatment options and significant number of relapses [4]. Microbiological diagnosis of brucellosis remains challenging, as isolation of the etiologic agent is hazardous and restricted to specialized laboratories, e.g., biosafety level 3. Thus, diagnosis relies mainly on serology. Treatment in farm animals is forbidden in many countries and vaccines are not always protective and safe for human health [5]. Eradication programs strictly follow test and slaughter/culling policy. Dogs are susceptible to *B. melitensis* and *B. suis* and could remain asymptomatic carriers for *B. abortus* [6,7,8]. Canine brucellosis caused by *B. canis* is manifested by late abortion and retention of fetal membranes in female dogs and orchitis, epididymitis and testicular atrophy in male dogs. Infected animals shed bacteria in body secretions viz. vaginal fluid, semen, saliva, nasal and ocular secretions, feces and milk and can transmit the infection directly through contact or indirectly via contamination of the environment [1,9,10]. Canine brucellosis is communicable to humans and other animal species and infections have been reported in different parts of the world [11]. Largely, brucellosis in dogs is considered as an under-estimated hazard to human health and animal welfare [12].

In Pakistan, brucellosis is considered endemic in ruminants and *B. abortus* and *B. melitensis* have been isolated from bovines and small ruminants, respectively [13,14,15]. Human brucellosis is well reported and has been described as a professional health hazard [16,17,18]. However, knowledge on the status of infection and possible epidemiological role of non-ruminant domestic animals (equine, canine, and feline) and wildlife in brucellosis remains scarce in the country [19,20,21,22].

A tremendous increase in popularity of keeping dogs as pets has been seen in Pakistani society over the last two decades [23]. Existence of a strong human–animal (dog) bond may pose a serious risk of transmission of brucellosis, especially among dog keepers. Serological tests that detect smooth lipopolysaccharide (LPS) of *Brucella* (*B. aboruts, B. melitensis* and *B. suis*) do not detect rough LPS (*B. canis* and *B. ovis*) [24]. Previous studies are limited to serological detection of livestock brucellosis in dogs [19]. Thus, we designed the current study to investigate the prevalence of antibodies against the smooth LPS antigen of *B. abortus* and *B. melitensis*, the rough LPS antigen of *B. canis* in dog sera, and the possible detection/differentiation of *Brucella* DNA at the species level to precisely identify the etiology and to determine related risk factors for the corresponding infection.

## 2. Results

A total of 37.6% (68/181) samples were found to be seropositive for canine brucellosis (*B. canis*) by slow agglutination test (SAT) and 4.9% (9/181) for livestock brucellosis (*B. abortus* and *B. melitensis*) by ELISA. The seroprevalence of *B. canis* was significantly higher in the dogs from Bahawalpur (63.8%, confidence interval (CI) 53.3–73.5) as compared to those from Faisalabad (9.2%, CI 4.1–17.3), Chi-square (χ^2^) = 56.55, *p* < 0.001. Using PCR, only one sample originating from the Faisalabad region was detected as positive for *B. abortus*.

Of the 94 serum samples collected from Bahawalpur, 60 (63.8%) were positive by SAT (Table 1) which subsequently tested negative by ELISA and real-time PCR. Among 87 serum samples originating from Faisalabad, eight (9.2%) were found positive by SAT and nine (10.3%) by ELISA. One ELISA positive sample amplified *B. abortus* DNA by real-time PCR. As no amplification was found for *B. melitensis*, we assumed livestock brucellosis was caused by *B. abortus* in these dogs. 

Location, age and body condition were the variables that showed significant (*p* < 0.05) association with *B. canis* in dogs. In the univariable analysis, dogs from Bahawalpur (odds ratio (OR) 17.43, CI 7.52–40.37), between 1–2 years of age (OR 3.96, CI 1.73–9.06), above two years of age (OR 3.09, CI 1.29–7.39) and with weak body condition (OR 2.73, CI 1.45–5.16) were found likely to test positive for *B. canis* (Table 1). In the multivariate analysis, location and age factors were found to be associated with *B. canis* prevalence. The model showed that dogs from Bahawalpur (OR 16.41, CI 6.99–38.53), between 1–2 years of age (OR 3.12, CI 1.19–8.15) and >2 years of age (OR 2.94, CI 1.06–8.17) were more likely to test positive for *B. canis* (Table 2). Sex, contact with other animals, presence of wounds, presence of ticks and external parasites, fever, and eye condition were excluded from the multivariate model at different steps as these variables did not show statistical association (*p* > 0.05) with infection (Table 1).

The presence of wounds (OR 4.26, CI 1.03–17.65) showed significant association (*p* < 0.05) with *B. abortus* prevalence in the univariate analysis. Location and contact with other animals could not be analyzed as no positive samples were found in Bahawalpur. All other variables did not show significant association (*p* > 0.05) to the infection, and hence multivariate analysis could not be performed (Table 3).

## 3. Discussion

Brucellosis remains a persistent zoonotic infection mainly caused by *B. abortus* and *B. melitensis* in ruminants in Pakistan and neighboring countries [25,26,27,28,29,30,31,32,33]. Canine brucellosis (*B. canis*) has also been reported in surrounding countries [34,35,36]. Faisalabad is the third largest city and is one of the leading districts in terms of daily milk production in the country. It bears a total of 2.7 million livestock heads; a mostly bovine population [37]. Brucellosis has been reported from Faisalabad in bovines, equines, camels and humans [38,39,40]. Bahawalpur is a relatively smaller city with a livestock population of 2.4 million heads, mostly small ruminants raised as nomadic/pastoral herds [37]. Only a few reports exist for brucellosis in bovines from Bahawalpur [38,40].

Serology is a main stay of brucellosis diagnosis and nonspecific reactions are not uncommon [41]. ELISA is a reliable diagnostic solution for livestock brucellosis (*B. abortus/B. melitensis/B. suis*) [42]. *B. canis* is of the rough LPS type and standard diagnostics for livestock brucellosis cannot be used. However, lower sensitivity of the antigen preparations of *B. canis* hampers diagnosis [43]. Real-time PCR assays may detect cases with negative serology [44]. No specific real-time assay for *B. canis* has been established yet. For livestock brucellosis, simultaneous serology and real-time PCR assays were applied to detect and differentiate *B. abortus* and/or *B. melitensis*. We used an in-house prepared *B. canis* antigen in SAT for the diagnosis of canine brucellosis. Besides SAT, a genus specific real-time assay was used.

The size of the dog population is unknown in Pakistan [45,46]. Dogs are mainly kept for watch purposes and to a lesser extent for companionship and fighting competitions [23]. Dogs kept at dairy farms/households in rural areas are used to guard the animals inside and outside of the dairy farms and during grazing. It is challenging to differentiate these rural household dogs from stray dogs as they often roam freely in the nearby countryside. Both of these types of dogs often have access to the rejected flesh from slaughterhouses, butcheries or municipal dumps, dead animal carcasses and remains of livestock, e.g. placentas or aborted fetal material, and also to kitchen leftovers [47,48,49].

The prevalence of antibodies to *B. canis* varied statistically significant (*p* < 0.05) among dogs from Faisalabad and Bahawalpur. Dogs from Bahawalpur were found to be more likely (OR 16.41, CI 6.99–38.53) to have canine brucellosis than those from Faisalabad (Table 1 and Table 2). An alarmingly high number of SAT positive dogs 63.8% (60/94) were found negative by PCR. This may be attributed to the persistence of infection as well as by intensive breeding with few preferred but *B. canis* infected males and vice versa [9,10]. The relatively higher number of *B. abortus* cases in Faisalabad indicated that these dogs had regular contact with *Brucella* antigen, and chronic persistence of infection in some of these stray dogs may be present too. Recently, DNA from *B. abortus* was detected in soil in Faisalabad where animals and humans lived in proximity [50]. The detection of *B. abortus* DNA in a seropositive stray dog confirms the actual presence of infection in the dog population of Faisalabad. No such proof existed for Bahawalpur. It is known that *Brucellae* from livestock are transmissible to animals living in close contact. The role of farm dogs as a host for bovine brucellosis and a source of re-infection at the farm level has already been confirmed [51,52,53,54]. Thus, counter measures must also include dogs having access to farms. This is also true for *B. canis* as infected dogs shed *Brucellae* in milk, urine, feces, nasal and ocular secretions, as well as in their saliva, and a risk for transmission to humans and other animals can also be assumed [55,56,57,58].

For *B. canis*, age was found to be associated (*p* < 0.05) with a higher risk of dogs older than one year of age (Table 1). The highest risk was found in 1–2 year old dogs. A similar pattern was observed in European and Iranian dogs [34,59]. Age might reflect repeated contact with *B. canis* excreting conspecifics. For *B. abortus*, such an association was found in Africa [60], but could not be confirmed in our study.

Dogs with weak body condition bared a 2.73 (1.45–5.16: 95% CI) times higher risk than dogs with normal body condition for canine brucellosis seropositivity. This may be attributed to weaker immune systems and failure to compete with healthier dogs. A non-significant association was found for *B. abortus* infection (Table 1 and Table 3).

The presence of wounds on the body indicated a 4.26 (1.03–17.65; 95% CI) times higher risk for being seropositive for livestock brucellosis (*B. abortus*) (Table 3). The meaning of this finding is not clear but may be connected to food acquisition. Based on our observations, these dogs feed on leftovers (placenta etc.) of bovines, fight with each other, get injured by physical barriers while entering feeding places or simply get hurt by people protecting farms or places where cadavers or discards are stored. Further studies can help to explain this finding.

A statistically non-significant (*p* > 0.05) association was found with the sex of the animals with seropositivity for both *B. canis* and *B. abortus*. A similar pattern was found in India but has remained unestablished in dogs from Jordan and Europe [59,61,62]. Similarly, a statistically non-significant association (*p* > 0.05) was found for tick and ecto-parasite infestation, fever and eye condition.

Although the number of dogs investigated is low, this study draws attention to the fact that brucellosis in stray dogs can be present. The low number of samples is due to the semi-wild lifestyle of the dog population, as blood sampling is simply often impossible and can be hazardous for the operator.

## 4. Materials and Methods

A total of 181 serum samples were collected from stray and working dogs based on convenient sampling: 87 serum samples were collected from Faisalabad and 94 from Bahawalpur districts in Punjab, Pakistan between December 2015–2016. Blood samples were collected under sterile conditions and serum was separated and kept at −20 °C until further analysis. The serum samples were sent to the Office International des Epizooties and National Reference Laboratory for Brucellosis at the Friedrich-Loffler Institute (FLI), Jena, Germany for serological and molecular diagnosis. All sera were tested by SAT using rough *B. canis* LPS antigen (FLI, Jena, Germany) as described by [63]. A titer >1:50 was considered as positive. The ID Screen^®^ Brucellosis serum indirect multi-species enzyme linked immunosorbent assay (ELISA) for the detection of anti-smooth LPS antibodies of *B. abortus* and *B. melitensis* was performed following the manufacturer’s recommendations (ID Vet, Garbels, France). Both districts were chosen as representative for different epidemiological settings of livestock brucellosis. Based on previous literature, we supposed that *B. melitensis* is prevalent in these districts although no isolates were available at the time of sample collection [64].

The sera were subjected to DNA extraction using the QIAamp DNA Mini Qiacube kit (Qiagen, Hilden, Germany) along with *E. coli* biomass as a negative control in each run. By using real-time PCR, molecular detection and differentiation of *Brucella* DNA (*B. abortus* and *B. melitensis*) was made [65]. For each run, positive controls of *B. abortus* (ATCC 23448) and *B. melitensis* (ATCC 23456) along with a no template control (NTC) of nuclease free water were included (see Table S1). PCR conditions were as follows: decontamination at 50 °C for 2 min, 1 cycle; initial denaturation at 95 °C for 10 min, 1 cycle; denaturation at 95 °C for 25 secs and 57 °C for 1 min for annealing and elongation of the primers, both for 50 cycles each. Samples showing cycle threshold (Ct) ≤38 were considered positive as based on in-house validation using camel sera [66].

For statistical analysis, the seroprevalence along with 95% exact binomial confidence interval (CI) was calculated. The Chi-square test (χ2) was used to determine significant differences. Furthermore, the data regarding different variables were subjected to univariable analysis to determine the association between the independent/explanatory variables obtained from the questionnaire and brucellosis seropositivity as the dependent/outcome variable. Odds ratio (OR), along with the respective CI, was calculated for different variables. All variables with the *p* value < 0.20 were included in the initial binary logistic regression model, and a backward stepwise approach was used to exclude the variable with the highest *p* value until all the confounders were removed [67]. The values of the Nagelkerke R^2^ (NR^2^) and Hosmer and Lemeshow Test (HL) were used to judge the fitness of the final model. Analysis was done using the SPSS software (IBM SPSS Statistics for Windows, Version 20.0).

## 5. Conclusions

Brucellosis in dogs is caused by *B. abortus* and *B. canis* in Faisalabad and Bahawalpur, Pakistan. *B. abortus* was confirmed by real-time PCR in dogs from Faisalabad but not in dogs from Bahawalpur. Geographical location seemed to play a role in the epidemiology of *B. canis* infection. Being older than one year of age and a weaker body condition were associated with *B. canis* but not with *B. abortus*. The presence of wounds on the body was associated with *B. abortus* infection only. Other factors such as sex, contact with other animals, ecto-parasite infestation, fever and eye condition were not associated with *B. canis* or *B. abortus* infection. Further studies may help in understanding the epidemiology of the infection. It becomes apparent that pet dogs and their owners have to be investigated to estimate the risk of human transmission. Humans in close contact with infected dogs should be tested for both *B. canis* and *B. abortus*/*B. melitensis*. Isolation and identification of *Brucellae* from clinically ill dogs and humans is important for molecular epidemiology. Up-to-date laboratory facilities and training is required for this purpose. Comprehensive studies may include all animals living near infected dogs and more than one diagnostic test should be used. Counter measures must include raising public awareness for canine brucellosis and routes of transmission to other animals and humans. Stray, wild and semi-wild canines must be included in brucellosis surveillance and eradication program. Although the study did not include clinically ill dogs, they should be included in future studies. Clinically healthy dogs may carry the infection sub-clinically, hence prior screening is necessary. To the best of our knowledge, this is the first study to test and report the presence of *B. canis* and *B. abortus* in dogs from Pakistan.

## Figures and Tables

**Table 1:** Univariate analysis of risk factors for canine brucellosis (*B. canis*).

Variable	Category	Positive/Tested	Prevalence, % (95% CI)	OR (95% CI)	*p* Value *
Location	Faisalabad	8/87	9.2 (4.1–17.3)	Ref	<0.001
	Bahawalpur	60/94	63.8 (53.3–73.5)	17.43 (7.52–40.37)	
Contact with animals	No	28/61	45.9 (33.1–59.2)	1.70 (0.90–3.19)	0.1
	Yes	40/120	33.3 (25–42.5)	Ref	
Sex	Male	44/123	35.8 (27.3–44.9)	Ref	0.468
	Female	24/58	41.4 (28.6–55.4)	1.27 (0.67–2.40)	
Age groups	<1 year	10/53	18.9 (9.4–32)	Ref	0.004
	1–2 years	35/73	47.9 (36.1–60)	3.96 (1.73–9.06)	
	>2 years	23/55	41.8 (28.7–55.9)	3.09 (1.29–7.39)	
Body condition	Weak	33/62	53.2 (40.1–66)	2.73 (1.45–5.16)	0.002
	Normal	35/119	29.4 (21.4–38.5)	Ref	
Wounds	No	40/120	33.3 (25–42.5)	Ref	0.1
	Yes	28/61	45.9 (33.1–59.2)	1.70 (0.90–3.19)	
Tick infestation	No	41/96	42.7 (32.7–53.2)	1.60 (0.87–2.95)	0.13
	Yes	27/85	31.8 (22.1–42.8)	Ref	
Ecto-parasites	No	47/116	40.5 (31.5–50)	1.43 (0.75–2.70)	0.275
	Yes	21/65	32.3 (21.2–45.1)	Ref	
Fever	No	57/152	37.5 (29.8–45.7)	Ref	0.965
	Yes	11/29	37.9 (20.7–57.7)	1.02 (0.45–2.31)	
Eye condition	Normal	52/132	39.4 (31–48.3)	3.25 (0.37–28.61)	0.515
	Red	15/43	34.9 (21–50.9)	2.68 (0.29–25.08)	
	Ulcer	1/6	16.7 (0.4–64.1)	Ref	
Total		68/181	37.6 (30.5–45.1)		

* *p* value < 0.05 considered as significant.

**Table 2:** Multivariate analysis of risk factors for canine brucellosis (*B. canis*).

Variable	Exposure Variable	Comparison	OR	95% CI	*p* Value
Location	Bahawalpur	Faisalabad	16.41	6.99–38.53	<0.001
Age group	1–2 years	<1 year	3.12	1.19–8.15	0.049
	>2 years	<1 year	2.94	1.06–8.17	

Model Fit: Nagelkerke R^2^ = 0.435, Hosmer and Lemeshow Test (χ = 4.004, *p* = 0.405).

**Table 3:** Univariate analysis of risk factors for livestock brucellosis (*B. abortus*).

Variable	Category	Positive/Tested	Prevalence % (95% CI)	OR (95% CI)	*p* Value *
Location	Faisalabad	9/87	10.3 (4.8–18.7)	-	-
	Bahawalpur	0/94	0 (0–3.8)	-	
Contact with animals	No	0/61	0 (0–5.9)	-	-
	Yes	9/120	7.5 (3.5–13.8)	-	
Sex	Male	8/123	6.5 (2.8–12.4)	3.97 (0.48–32.48)	0.199
	Female	1/58	1.7 (0–9.2)	Ref	
Age groups	<1 year	1/53	1.9 (0–10.1)	Ref	0.278
	1–2 years	6/73	8.2 (3.1–17)	4.66 (0.54–39.89)	
	>2 years	2/55	3.6 (0.4–12.5)	1.96 (0.17–22.31)	
Body condition	Weak	1/62	1.6 (0–8.7)	Ref	0.167
	Normal	8/119	6.7 (2.9–12.8)	4.40 (0.54–35.98)	
Wounds	No	3/120	2.5 (0.5–7.1)	Ref	0.046
	Yes	6/61	9.8 (3.7–20.2)	4.26 (1.03–17.65)	
Tick infestation	No	6/96	6.3 (2.3–13.1)	1.82 (0.44–7.52)	0.407
	Yes	3/85	3.5 (0.7–10)	Ref	
Ecto-parasites	No	6/116	5.2 (1.9–10.9)	1.13 (0.27–4.67)	0.869
	Yes	3/65	4.6 (1–12.9)	Ref	
Fever	No	7/152	4.6 (1.9–9.3)	Ref	0.605
	Yes	2/29	6.9 (0.8–22.8)	1.53 (0.30–7.79)	
Eye condition	Normal	7/132	5.3 (2.2–10.6)	1.15 (0.23–5.75)	0.986
	Red	2/43	4.7 (0.6–15.8)	Ref	
	Ulcer	0/6	0 (0–45.9)	-	
Total		9/181	5 (2.3–9.2)		

* *p* value < 0.05 considered as significant.

## Supplementary Materials

The following are available online at https://www.mdpi.com/2076-0817/8/4/294/s1, Table S1: Primer and probes sequence for real-time PCR.
