# Supplementary material for: Serological and Molecular Investigation of Brucella Species in Dogs in Pakistan
Source: Pathogens. 2019 Dec 13;8(4):294. doi: 10.3390/pathogens8040294 (PMC6963446; doi:10.3390/pathogens8040294)
Supplement: Supplementary file 1 [file pathogens-08-00294-s001.pdf]

Table S1: Primer and probes sequence for real-time PCR

Detection	Primers/Probes	Sequence (5'-3')	Reference
Brucella (bcsp31)	Forward Primer	GCTCGGTTGCCAATATCAATGC	[65]
	Reverse Primer	GGGTAAAGCGTCGCCAGAAG	
	Probe	FAM-AAATCTTCCACCTTGCCCTTGCCATCA-BHQ	
B. abortus (IS711)[1]	Forward Primer	GCGGCTTTTCTATCACGGTATTC	
	Reverse Primer	CATGCGCTATGATCTGGTTACG	
	Probe	HEX-CGCTCATGCTCGCCAGACTTCAATG-BHQ	
B. melitensis (IS711)[2]	Forward Primer	AACAAGCGGCACCCCTAAAA	
	Reverse Primer	CATGCGCTATGATCTGGTTACG	
	Probe	Cy5-CAGGAGTGTTTCGGCTCAGAATAATCCACA-BHQ	

[1]*alkB B. abortus* biotype 1 (strain 544/*ATCC 23448), [2]BMEI1162 *B. melitensis* biotype 1 (strain 16M/*ATCC 23456), *American Type Culture Collection
